# Effects of ultrasound-guided lumbar-sciatic nerve block and epidural anesthesia on the levels of IL-6, IL-8, TNF-α and coagulation factors in peripheral blood of elderly patients after hip arthroplasty

**DOI:** 10.5937/jomb0-35847

**Published:** 2022-10-15

**Authors:** Zhi Huang, Yan Cai, Yunfei Yang, Jin Shi, Xuya Zhao, Haise Mo, Qingfan Zeng

**Affiliations:** 1 The Affiliated Hospital of Guizhou Medical University, Department of Interventional Radiology, Guiyang, People Republic China; 2 Guizhou Medical University, School of Basic Medical Science, Guiyang, People Republic China; 3 The Affiliated Hospital of Guizhou Medical University, Ultrasonic Center, Guiyang, People Republic China; 4 Guizhou Medical University, Institute of Image, Guiyang, People Republic China; 5 The Affiliated Hospital of Guizhou Medical University, Guiyang, Department of Anesthesiology, Guizhou, People Republic China; 6 The Affiliated Baiyun Hospital of Guizhou Medical University, Guiyang, Department of Anesthesiology, Guizhou, People Republic China

**Keywords:** lumbar slave-sciatic nerve block, epidural anesthesia, inflammatory factor, coagulation factor, blok lumbalnog slave-išijadičnog nerva, epiduralna anestezija, inflamatorni faktor, faktor koagulacije

## Abstract

**Background:**

To investigate the effects of ultrasound-guided lumbar-sciatic nerve block and epidural anesthesia on the levels of inflammatory factors such as Interleukin-6 (IL6), Interleukin-8 (IL-8), Tumor necrosis factor-a (TNF-α) and coagulation factors in peripheral blood of elderly patients after hip arthroplasty to provides reference value for the choice of intraoperative anesthesia.

**Methods:**

96 elderly patients underwent hip arthroplasty in our hospital from March 2018 to December 2019 were selected and divided into ultrasound-guided lumbar-sciatic nerve block group (group A) and epidural anesthesia group (group B) randomly , there were 48 cases in each group. The onset time of intraoperative anesthesia, postoperative hemodynamic indexes, pain score, inflammatory factors and blood coagulation factor levels were compared between group A and group B.

**Results:**

It was proved that: (1) The onset time of sensory block and motor block in group B were shorter compared with group A, and the maintenance time of anesthesia was prolonged (P<0.05); (2) Compared with group A, visual analogue scale (VAS) score of group B patients after operation was lower (P<0.05); (3) The systolic blood pressure (SBP) and diastolic blood pressure (DBP) of group B were higher than group A (P<0.05) ) at T1 and T2, while the comparison of SBP and DBP between groups was not statistical difference at T3 and T4 (P>0.05); (3) Compared with group A, the levels of TNF, IL-8and IL-6 in peripheral blood of group B decreased after T2, T3 and T4 (P<0.05); (4) Statistical difference in plasma factor V activity (FV:C), coagulation factor VIII activity (FVIII:C) and fibrinogen (FIB) levels were showed between groups A and B at T2, T3 and T4 (P<0.05) with significantly lower values in group B compared to group A(P<0.05). (5) The half-year mortality rates of patients in two group were 5.56% and 8.33% respectively. There was no significant difference between group A and group B (P>0.05).

**Conclusions:**

Compared with epidural anesthesia, lumbarsciatic nerve block is showed significantly lower values in concentration of peripheral blood coagulation factors and inflammatory factors after surgery, thereby alleviating postoperative hypercoagulability and inflammation.

## Abstract (sr)

**Uvod:**

Cilj je bio da se istraže efekti ultrazvučno vođene blokade lumbalnog išijadičnog nerva i epiduralne anestezije na nivoe inflamatornih faktora kao {to su interleukin-6 (IL-6), interleukin-8 (IL-8), faktor nekroze tumora-a (TNFa) i faktori koagulacije u perifernoj krvi starijih pacijenata posle artroplastike kuka kako bi se dobile referentne vrednost za izbor intraoperativne anestezije.

**Metode:**

96 starijih pacijenata kojima je urađena artroplastika kuka u na{oj bolnici od marta 2018. do decembra 2019. godine odabrano je i podeljeno u ultrazvučno vođenu grupu lumbalnog išijanog nervnog bloka (grupa A) i grupu epiduralne anestezije (grupa B) nasumično, bilo je 48 slučajeva u svaka grupa. Vreme početka intraoperativne anestezije, postoperativni hemodinamski indeksi, skor bola, inflamatorni faktori i nivoi faktora koagulacije krvi upoređivani su između grupe A i grupe B.

**Rezultati:**

Utvrđeno je da je: (1) Vreme nastanka senzornog bloka i motornog bloka u grupi B je kraće u poređenju sa grupom A, a vreme održavanja anestezije je produženo (P<0,05); (2) U poređenju sa grupom A, skor na vizuelnoj analognoj skali (VAS) pacijenata grupe B posle operacije je bio niži (P<0,05); (3) Sistolni krvni pritisak (SBP) i dijastolni krvni pritisak (DBP) grupe B bili su vi{i od grupe A (P<0,05) na T1 i T2, dok poređenje SBP i DBP izme|u grupa nije bilo statističke razlike u T3 i T4 (P>0,05); (3) U poređenju sa grupom A, nivoi TNF-a, IL-8 i IL-6 u perifernoj krvi grupe B su smanjeni nakon T2, T3 i T4 (P<0,05); (4) Statistička razlika u aktivnosti faktora V plazme (FV:C), aktivnosti faktora koa gulacije VIII (FVIII:C) i nivoa fibrinogena (FIB) prikazana je izme|u grupa A i B na T2, T3 i T4 (P<0,05) sa značajno niže vrednosti u grupi B u odnosu na grupu A (P<0,05). (5) Polugodišnji mortalitet pacijenata u dve grupe iznosio je 5,56%, odnosno 8,33%. Nije bilo značajne razlike između grupe A i grupe B (P>0,05).

**Zaključak:**

U poređenju sa epiduralnom anestezijom, blok lumbalno-šijadičnog nerva pokazuje značajno niže vrednosti koncentracije faktora koagulacije periferne krvi i inflamatornih faktora posle operacije, čime se ublažava postoperativna hiperkoagulabilnost i inflamacija.

## Introduction

Common orthopedic diseases in the elderly include femoral head necrosis, hip fracture, osteoarthritis or rheumatoid arthritis, etc [1]. Hip arthroplasty can relieve the pain of patients, improve the motor function of the joints, thereby improving the quality of life, and is considered to be a treatment with better curative effects [2]. With the aging of the population, the design of prosthesis materials [3]. and the continuous development of medical technology, the number of elderly patients who need hip replacement surgery is increasing. Replacement surgery's, which often take a relatively long time and may compared with large amount of bleeding, is performed under general anesthesia alone or combination with local anesthesia.

The elderly have poor tolerance to surgery and anesthesia, and the risk of anesthesia during the perioperative period is high [4]. Choosing inappropriate anesthesia methods will not only affect the patient's postoperative recovery, but even threaten the patient's life. The elderly are mostly associated with basic diseases such as hypertension, and the operation of the replacement surgery is limited, and most patients do not need to use general anesthesia [5]. Ultrasound-guided nerve block and epidural anesthesia are local anesthesia methods which are used more [6].

Under normal circumstances, the body is in a state of normal coagulation function, and the anticoagulation system and procoagulant system in the blood are in a dynamic balance [7]. When various factors are combined, the balance will be broken. When in a state of hypercoagulability, blood clots are prone to form in the blood vessels, triggering a series of adverse reactions [8]. For elderly patients undergoing hip replacement surgery, affected by age, the patient's various functions gradually decline, and the blood coagulation function declines. When elderly patients undergo hip replacement surgery, anesthesia punctures, surgical operations, and surgical trauma can cause damage to the patient's vascular endothelium. When the vascular intima is damaged, a large number of inflammatory factors are released, causing vasospasm and activating the blood coagulation system in the body. Affected by ages, the hypercoagulable state after the operation increases the risk of thrombosis. After thrombosis, the blood vessels become narrow and the blood volume of the distal return heart becomes less, causing blood stasis, and the patient's lower limbs are swollen and painful. When the thrombus falls off, it will travel along with the blood circulation, and it is easy to block the pulmonary artery and other places, and the patient will have difficulty breathing, which even causes life-threatening.

Currently, there is no prospective study on the effect of these two local anesthetics on the levels of peripheral blood inflammatory factors in elderly patients undergoing hip replacement surgery. This article investigates the effect of ultrasound-guided lower back-sciatic nerve block and epidural anesthesia on the inflammatory factorsInterleukin-6 (IL-6), Interleukin-8 (IL-8), Tumor necrosis factor-α(TNF-α) and the clotting factor levels in the peripheral blood of patients underwent hip replacement surgery.

## Materials and methods

### Patients and study design

96 elderly patients underwent hip arthroplasty in our hospital from March 2018 to December 2019 were randomly selected. Reasons for hip replacement: 60 cases of femoral neck fracture, 20 cases of osteoarthritis of hip, 16 cases of femoral head necrosis. Inclusion criteria: (1) Aged 65 or older; (2) The patients were all grade II-III according to the classification system of American Society of Anesthe siologists (ASA); (3) Blood coagulation function was normal. Exclusion criteria: (1) Peripheral nerve injuried; (2) Allergy or contraindications to local anesthetics used in this study; (3) Patients with spine diseases; (4) Incomplete clinical data. All the research subjects and their family members agreed and an informed consent form was signed. The selected patients were divided into two groups according to the random number table method, and there were 72 patients in each group. During operation, the patients of two groups were treated with different anesthesia methods. Group A received continuous epidural anesthesia, and group B received ultrasound-guided lumbarsciatic nerve block. There were 20 males and 28 females in group A who were 65-79 years old with age of (70.74±3.86), and group B included 22 males and 26 females who were 65-80 years old with age of (70.81±3.92) (Table 1).

**Table 1:** Characteristic information about the patients. 1 median and range, 2 mean and SD, ^*^BMI is the bdy mass index.

Characteristics	Group A<br>n=48	Group B<br>n=48	P value
Age (years)^1^	70.74±3.86	70.81±3.92	0.715
Gender (n, %)			0.232
Male	20 (41.67%)	22 (45.83%)	
Female	28(	26(	
BMI* (kg/m^2^)^2^	22.16±2.01	21.82±2.05	0.414
Operation time<br>(min)	About<br>90 minutes	About<br>90 minutes	0.999

### Methods of anesthesia

All patients received no drug treatment and an 8-hour fasting before the operation. Routinely monitor vital signs of patients such as SBP, DBP, heart rate (HR) and other indicators. Dexmedetomidine (Sichuan Guorui Pharmaceutical, China) was continuously pumped intravenously, with a loading dose of 0.5 μg·kg^-1^ for 10∼15 minutes, and then maintained infusion of 0.3 μg·kg^-1^·h^-1^. Invasive arterial blood pressure was monitored by puncture and catheterization of radial artery under local anesthesia.

Patients in group A underwent epidural anesthesia. Epidural puncture was performed at L2-3, the epidural catheter was introduced to the head, and 3 mL of 2% lidocaine (Zhaohui pharmaceutical, China) was injected through the catheter after lying supine. After observing no adverse reaction, inject 0.5% ropivacaine (AstraZeneca AB, Sweden) 8∼12 mL in batches.

Patients in group B underwent lumbar plexussciatic nerve block under ultrasound guidance (Sonosite EDGE, USA). After sedated, the patient was placed side up, lying on his side, with hips and knees bent. The puncture needle was withdrawn and there was no return of blood, 0.4% ropivacaine (Astra-Zeneca AB, Sweden) was injected after puncture, and the ultrasound image was observed 3 minutes after the injection, and the remaining drugs were injected after no adverse reactions. 30 mL was injected into the lumbar plexus and the sciatic nerve 20 mL. If the two groups of patients had mild pain and discomfort during the operation, sufentanil (Yichang Human well Pharmaceutical, China) 5∼10 μg was injected intravenously.

### Observation indicators

Monitor and record the changes in HR, SBP and DBP of patients in two groups entering the operating room (T_0_), postoperative 0h (T_1_), postoperative 24h (T_2_), postoperative 48h (T_3_) and postoperative 72h (T_4_). After the anesthesia was completed, the onset and maintenance time of sensory block and motor block were recorded. Pain score: at 24h (T_b_) after preoperative (T_a_), 48h (T_c_) after surgery and 72h (Td) after surgery, the visual analogue scale (VAS) [9] was used to evaluate the pain conditions of patients in two groups. 0-10 points, 0 means no pain, 10 means severe pain; mild pain, tolerable: 1-3 points; moderate pain, still tolerable: 4-6 points; severe pain, unbearable: 7-10 points.

### Observation indicators

At least 14 mL and maximally 20 mL citrated blood samples were obtained from the median cubital vein of patients in groups A and B, one tube of anticoagulation, one tube of non-anticoagulation. After sampling, the blood was centrifuged twice (15 min at 2500 g and 5 min at 10000 g, room temperature) to collect the corresponding serum and plasma at T_0_, T_2_, T_3_, and T_4_.

The enzyme-linked immunosorbent assay (ELISA) was used to detect concentrations of inflammatory factors include TNF-α, IL-8 and IL-6 in serum. The samples were tested for the level of FV:C, FVIII:C,and fibrinogen in the plasma by a one-stage APTT-based coagulometric method using the CA-6000 automatic blood coagulation system (Sysmex, Kobe, Japan).The relevant reagents were purchased from Siemens (Sysmex, Marburg, Germany). The re sults are presented as % (FV: C, FVIII: C), and g/L (FIB).

### Statistical analysis

The SPSS 24.0 software package was used. After of collected data had been checked, Measurement data was expressed as mean ± standard deviation (x̄±S) after normal distribution test, and two independent sample non-parametric tests were used for analysis between groups; count data was recorded as rate (%), the comparison between two groups was analyzed by chi-square test. All-cause mortality and survival were analyzed using Kaplan Meier survival curves, and the log-rank test for significance. The *P* value <0.05 was considered as statistical significantly. GradpadPrism 7.0 software package were used for mapping.

## Results

### Patient populations

Median age of patients in group Awas 70.74 years old, and of patients in group B was 70.81 years old. The proportion of male patients was 41.67% (20 cases) in group A and 45.83% in group B (22 cases). Average BMI of patients in group Awas 22.16±2.01, and of patients in group B was 21.82±2.05.There was no significant difference between two groups in age, sex ratio, body mass index (BMI) and operation time (*P*>0.05) (Table 1).

### Comparison of anesthesia onset and pain scores between two groups

**Figure 1:**
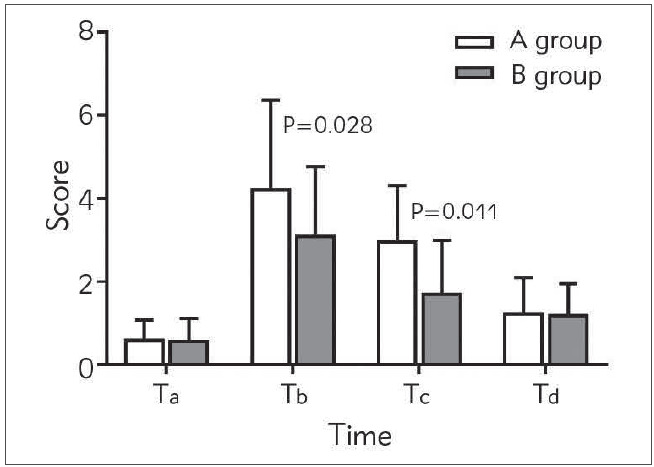
Comparison of pain scores between the patients in two groups at different time points.

Two different anesthesia methods, after the administration was completed, compared with group A, the onset time of sensory block and motor block of group B was shorter, and the duration of anesthesia was prolonged (*P*<0.05). There was no significant difference between two groups in VAS scores before operation (T_a_) (*P*>0.05). Compared with group A, the VAS score of group B patients decreased (*P*<0.05) at 24h (T_b_), 48h (T_c_) and 72h (T_d_) after operation (Table 2 and Figure 1).

**Table 2:** Comparison of onset time and maintenance time after anesthesia. aP<0.05, compared with group A.

Group	Sensory block		Motor block	
	Effective time	Hold time	Effective time	Hold time
A	11.71±2.15	350±23.45	13.89±2.50	285±18.50
B	9.28±1.71^a^	390.11±26.71^a^	12.15±2.15^a^	300.18±28.75^a^
*P*	0.002	0.009	0.025	0.014

### Comparison of hemodynamics between patients in two groups at different time points

**Figure 2:**
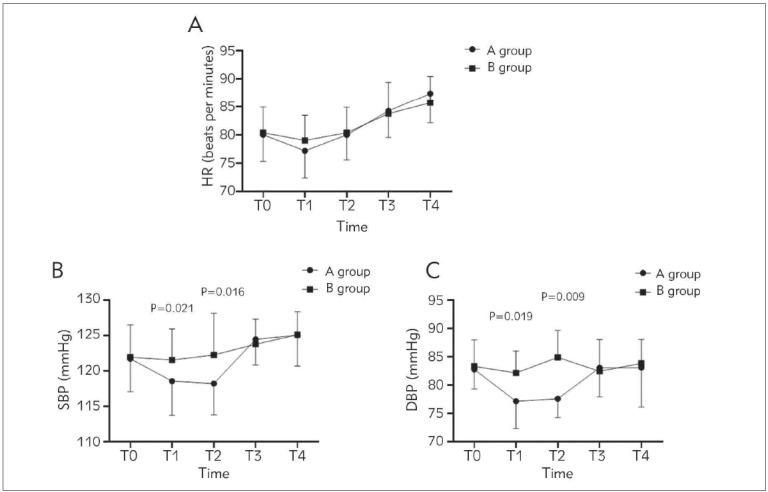
Comparison of hemodynamics between the two groups of patients at different time points.

Comparing the changes of the hemodynamic indexes HR, SBP and DBP of patients in two groups after operation, there were varying degrees of fluctuations. HR, SBP, and DBP at T_0_ as well as HR at T_1_-T_4_ of two groups showed no significant difference (*P*>0.05). The SBP and DBP of group B were higher than those of group A (*P*<0.05) at T_1_ and T_2_, but no significant difference was observed between T_3_ and T_4_ in SBP and DBP (*P*>0.05) (Figure 2).

### The levels of inflammatory factors IL-6, IL-8 and TNF-α in peripheral blood of patients in two groups

The concentration of IL-6, IL-8 and TNF-α in patients peripheral blood of two groups were monitored before and after anesthesia. No significant difference exhibited between groups in IL-6, IL-8 and TNF-α at T_0_ before operation (*P*>0.05). The levels of IL-6, IL-8 and TNF-α showed an upward trend, and began to decline at T_4_ after T_2_ and T_3_ in two groups. Compared with group A, the levels of IL-6, IL-8 and TNF-α in peripheral blood of group B decreased (*P*<0.05) after T_2_, T_3_ and T_4_ (Table 3).

**Table 3:** Comparison of the concentration of inflammatory factors (IL-6, IL-8 and TNF-α) in the peripheral blood of patients in groups A and B. T0: entering the operating room; T1: postoperative 0h; T2: postoperative 24h; T3: postoperative 48h; T4: postoperative 72h.<br>^a^P<0.05, compared with group A at the same time point.

Indicator	Group	T0	T2	T3	T4
IL-6<br>(pg/mL)	A	97.13±9.25	140.57±13.29	162.17±15.39	138.41±16.24
	B	98.48±9.89	132.04±10.58^a^	154.33±13.45^a^	128.35±15.67^a^
P		0.069	0.027	0.012	0.028
IL-8<br>(pg/mL)	A	254.71±17.56	315.81±23.47	330.56±28.63	300.73±27.13
	B	256.32±16.94	290.68±22.15^a^	310.63±27.25^a^	289.59±25.49^a^
P		0.061	0.009	0.001	0.038
TNF-α<br>(ng/mL)	A	0.68±0.15	1.05±0.18	1.36±0.23	1.18±0.15
	B	0.70±0.17	0.83±0.14^a^	1.12±0.18^a^	1.01±0.16^a^
P		0.078	0.016	0.024	0.043

### Comparison of coagulation function between the two groups

The data showed no significant difference in plasma FV: C, FVIII: C, and FIBat T_0_ before operation between groups (*P*>0.05). At the three time points of T_2_-T_4_ postoperatively, FV: C, FVIII: C and FIB increased over time. Compared with group A, plasma FV: C, FVIII: C and FIB in group B decreased (all *P*<0.05) at T_2_, T_3_ and T_4_ after operation (Table 4).

**Table 4:** Comparison of coagulation factors FV and FVIII and fibrinogen activities and thrombin time test between groups A and B. T0: entering the operating room; T1: postoperative 0h; T2: postoperative 24h; T3: postoperative 48h; T4: postoperative 72h. ^a^ *P*<0.05, compared with group A at the same time point.

Indicator	Group	T_0_	T_2_	T_3_	T_4_
FV:C (%)	A	115.21±15.63	135.64±19.88	143.77±16.23	157.93±20.34
	B	117.18±17.84	123.74±16.55^a^	128.81±17.45^a^	135.28±18.15^a^
*P*		0.058	0.032	0.011	0.007
FVIII:C (%)	A	107.19±10.13	130.27±13.45	143.64±21.26	168.24±25.47
	B	105.63±11.78	113.58±15.18^a^	120.56±14.15^a^	124.16±13.29^a^
*P*		0.126	0.016	0.005	0.001
FIB (g/L)	A	3.13±1.43	3.75±1.62	4.18±1.34	4.73±1.25
	B	3.07±1.50	3.21±1.75^a^	3.43±1.28^a^	3.56±1.07^a^
*P*		0.089	0.037	0.003	0.012

### Comparison of survival rates of the two groups

**Figure 3:**
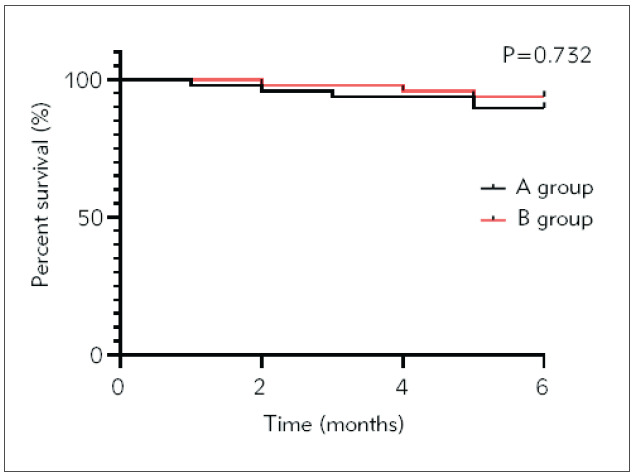
Comparison of the survival rate of the patients in two groups after half a year.

The patients in two groups were followed up for 6 months, and the survival status of the patients was recorded. The fatality rates of two groups were 5.56% and 8.33% at the end of the follow-up. The fatality rate of group A was slightly higher than that in group B. The two groups all had a high fatality rate. There was no significant differences between two groups (*P*>0.05) (Figure 3).

## Discussion

With the development of anesthesiology, medical workers have gradually realized that inappropriate anesthesia methods can not only cause instability of patients during surgery, but most importantly, increase the risk of postoperative complications and reduce the clinical value of replacement surgery. In application of hip surgery anesthesia, there are many controversies about the anesthesia effect of the two anesthesia methods, nerve block and epidural anesthesia, and the differences in postoperative complications [10]. In the past choice of replacement anesthesia, epidural anesthesia is a kind of intraspinal anesthesia which is simple to operate and has a good perioperative analgesic effect, and it is used by most clinicians [11]. However, the sympathetic nerve of the patient is blocked segmentally after anesthesia, and the patient is prone to drop in blood pressure during the operation. In order to maintain the stability of intraoperative blood pressure, fluid supplementation is often used to expand the volume, which may change the levels of inflammatory factors and coagulation factors in the peripheral blood of patients after surgery.

Nerve block, refers to the injection of local anesthetics around the nerve trunk, is an important method of local anesthesia. When performing hip replacement surgery, a combined block of the lumbar plexus and sciatic nerve can be implemented, which is simple to operate and has a definite anesthetic effect. The traditional blind probing method is to find the puncture site by the anesthesiologist based on clinical experience and the subjective expression of the patient. The location is extremely inaccurate causing nerve damage or local anesthetic poisoning, to limite clinical application. In recent years, under the guidance of ultrasound, the positioning accuracy of the puncture point has been greatly improved [12]. The process of local anesthetic injection and diffusion can be observed at any time, and dose can be adjust individually.

Evaluation of the overall clinical benefit of anesthesia can be divided into three aspects, namely anesthesia effect, stable hemodynamic status and fewer complications of anesthesia after surgery. Studies have shown that combined lumbar plexus-sciatic nerve block for patients undergoing joint surgery can meet the anesthesia requirements of the operation, and the patient’s perioperative hemodynamics can be maintained at a relatively stable level [13]. Our study compared the effects of epidural anesthesia (group A) and ultrasound-guided lumbar plexus-sciatic nerve block (group B) were compared. The results showed that the onset time of sensory block and motor block of group B was shorter, and the duration of anesthesia was prolonged. Compared with group A, the VAS score of group B patients decreased at 24h, 48h, and 72h after operation, which means ultrasound-guided lumbar plexus-sciatic nerve block has better advantages in terms of anesthesia effect and stable hemodynamic status.

The influence of different anesthesia methods on the coagulation function of patients after surgery showed controversy indifferent studies [14] [15]. The two groups of anesthesia methods used in this study may prevent postoperative blood coagulation, reduce the risk of thrombosis, and may have no effect on the patient's coagulation function. The results of our study showed that FV:C, FVIII:C and FIB of the two groups showed an upward trend at three time points from 24h to 72 after surgery. Compared with group A, FV: C, FVIII: C and FIB in the plasma of group B patients decreased. FIB can reflect whether the human body can automatically complete the process of hemostasis. The results show that ultrasound-guided lumbar plexus-sciatic nerve block can reduce the activity of blood coagulation factors and relieve postoperative hypercoagulability.

Patients generally have stress immune response after surgery. Many studies have reached different conclusions about the effect of anesthesia type and postoperative analgesia on the immune response to surgically induced stress [16] [17] [18]. A meta-analysis compared the effects of epidural anesthesia and general anesthesia on postoperative natural killer T cell function in patients [19]. The results showed that different anesthesia methods seem to have no significant effect on these cell functions. Another study reported that 54 patients requiring extensive abdominal surgery were treated with epidural anesthesia, which can prevent pressure-induced dysfunction of pro-inflammatory lymphocytes during the perioperative period [20]. Our study found that the levels of IL-6, IL-8 and TNF-α in two groups showed an upward trend 24h-48h after surgery, and began to decline at 72h after surgery. Compared with group A, the levels of IL-6, IL-8, and TNF-α in peripheral blood of group B patients decreased at the three time points of 24h, 48h and 72h after operation. The results indicate that ultrasound-guided lower back-sciatic nerve block may inhibit postoperative inflammation in elderly patients undergoing hip replacement. Patients in groups A and B were followed up for 6 months, and the survival status of the patients was recorded. Although no statistically significant difference was showed between two groups in postoperative mortality, our data provided a good comparison of postoperative acute inflammation levels between the two groups after hip arthroplasty.

In summary, this study illustrate that ultrasound-guided lower lumbar plexus-sciatic nerve block has a better anesthesia effect for elderly patients undergoing hip replacement surgery, reducing concentration of peripheral blood coagulation factors and inflammatory factors after surgery, thereby alleviating Postoperative hypercoagulability and inflammation.

## Dodatak

### Funding

The Special Funds for the Central Government to Guide Local Science and Technology Development [grant no. QKZYD(2019)4008], The Science and Technology Department of Guizhou Province (Basic Science and Technology Cooperation [2017]1149], The technology project of the baiyun district of guiyang city 2019 -36 Guizhou Science and Technology Planning Project Guizhou Science and Technology Integration foundation -ZK[2021] General 489 Guizhou Provincial Science and Technology Department project 2021 PhD Research Start-up Fund gyfybsky-2021-26.

### Conflict of interest statement

All the authors declare that they have no conflict of interest in this work.
